# Supplementary material for: Modeling the Competition between Misfolded Aβ Conformers That Produce Distinct Types of Amyloid Pathology in Alzheimer’s Disease
Source: Biomolecules. 2022 Jun 24;12(7):886. doi: 10.3390/biom12070886 (PMC9313290; doi:10.3390/biom12070886)
Supplement: Supplementary file 1 [file biomolecules-12-00886-s001.zip › biomolecules-1763667-supplementary.pdf]

Supplementary Materials

Modeling the competition between misfolded Aβ conformers that produce distinct types of amyloid pathology in Alzheimer's disease

Guilian Xu[1-3], Susan Fromholt[1-3], David R. Borchelt[1-3]

[1]Department of Neuroscience, College of Medicine, University of Florida, Gainesville, FL 32610, USA.
[2]Center for Translational Research in Neurodegenerative Disease, College of Medicine, University of Florida, Gainesville, FL 32610, USA.
[3]McKnight Brain Institute, College of Medicine, University of Florida, Gainesville, FL 32610, USA.

Table S1. Data for individual animals from PrP.MoAβ/PS1 mice injected with seeding homogenates.

First Passage in PrP.MoAβ/PS1(Cr) mice					
Animal ID#	Sex	Seed Source	Age Harvested	Pathology Type	Score
0266885-2	M	None	9	None	-
0334921-1	M	None	10	None	-
0334921-2	M	None	10	None	-
0336477-3	F	None	10	None	-
0656952-1	M	None	12	None	-
0850873-1	M	PrP.APPsi (Df)	12	Df	++
0850872-1	F	PrP.APPsi (Df)	12	Df	+++
0884222-1	M	PrP.APPsi (Df	12	Df	++
0711779-4	F	Tet.MoAβ (Df)	9	Df	++
0711783-2	M	Tet.MoAβ (Df)	12	Df	+++
0973439-3	F	Tet.MoAβ (Df)	10	Df	+++
0973441-1	M	Tet.MoAβ (Df)	10	Df	+++
0793910-1	M	PrP.HuAβ/PS1 (Cr)	12	M	+
0874270-1	M	PrP.HuAβ/PS1 (Cr)	10	Cr	+
0879086-1	M	PrP.HuAβ/PS1 (Cr)	12	Cr	+
0879086-2	M	PrP.HuAβ/PS1 (Cr)	12	Cr	+
0879087-2	F	PrP.HuAβ/PS1 (Cr)	12	Cr	+
0720151-2	F	PrP.MoAβ/PS1 (Cr)	12	Cr	+
0706695-3	M	PrP.MoAβ/PS1 (Cr)	9	Cr	+
0864798-1	M	PrP.MoAβ/PS1 (Cr)	12	Cr	+
1025434-1	M	PrP.MoAβ/PS1 (Cr)	12	Cr	-
1025434-2	M	PrP.MoAβ/PS1 (Cr)	12	Cr	+
1025434-3	M	PrP.MoAβ/PS1 (Cr)	12	Cr	+

Table S1 (continued). Data for individual animals from PrP.HuAβ/PS1 mice injected with seeding homogenates.

First Passage in PrP.HuAβ/PS1(Cr) mice					
Animal ID#	Sex	Seed Source	Age Harvested	Pathology Type	Score
1004005-1	F	None	5	Cr	+
1004005-2	F	None	5	Cr	+
0574005-2	M	None	6	Cr	+
0862093-3	M	PrP.APPsi (Df)	6	Cr	+
0862093-4	M	PrP.APPsi (Df)	6	Cr	++
0862082-1	M	PrP.APPsi (Df)	6	Cr	+
0862087-1	F	PrP.APPsi (Df)	6	Cr	++
0871377-1	M	Tet.MoAβ (Df)	6	M	+
0871377-4	M	Tet.MoAβ (Df)	6	M	++
0871376-1	F	Tet.MoAβ (Df)	6	M	++
0879926-3	M	Tet.MoAβ (Df)	6	M	++
0879926-4	M	Tet.MoAβ (Df)	6	M	++
0793904-1	M	PrP.HuAβ/PS1 (Cr)	6	Cr	+
0793904-2	M	PrP.HuAβ/PS1 (Cr)	6	Cr	+
0793904-4	M	PrP.HuAβ/PS1 (Cr)	6	Cr	+
0808994-1	M	PrP.HuAβ/PS1 (Cr)	6	Cr	+
0808994-3	M	PrP.HuAβ/PS1 (Cr)	6	Cr	+
0808994-4	M	PrP.HuAβ/PS1 (Cr)	6	Cr	++
0808993-3	F	PrP.HuAβ/PS1 (Cr)	6	Cr	+
0808996-1	M	PrP.HuAβ/PS1 (Cr)	6	Cr	+
0808996-2	M	PrP.HuAβ/PS1 (Cr)	6	Cr	+
0879931-1	M	PrP.MoAβ/PS1 (Cr)	6	Cr	+
0879931-2	M	PrP.MoAβ/PS1 (Cr)	6	Cr	+
0879931-4	M	PrP.MoAβ/PS1 (Cr)	6	Cr	+
0879931-5	M	PrP.MoAβ/PS1 (Cr)	6	Cr	+
0879930-1	F	PrP.MoAβ/PS1 (Cr)	6	Cr	+
0896402-1	M	PrP.MoAβ/PS1 (Cr)	6	Cr	+
0896402-2	M	PrP.MoAβ/PS1 (Cr)	6	Cr	+

Table S2. Data for individual animals from serial passaging experiments.

Animal ID #	Sex	Passage History (host strain)	Age Harvested	Last Recipient Pathology Type at Harvest	Pathology Burden Score
0898272-1	F	Tet.MoAβ to PrP.APPsi to PrPHuAβ/PS1 (Df to Df to Cr)	6	M	++
0898279-2	M		6	M	++
0898279-4	M		6	M	++
0898276-1	F		6	M	++
0905961-3	F		6	M	++
0905960-2	F		6	M	++
0898273_1	F	Tet.MoAβ to PrP.APPsi to PrP.APPsi (Df to Df to Df)	12	Df	+++
0898274_1	M		12	Df	+++
0951046_1	F		12	Df	+++
1052846-1	M	Tet.MoAβ to PrP.APPsi to PrP.MoAβ/PS1 (Df to Df to Cr)	12	Df	+++
1052845-1	F		12	Df	+++
1052847-1	F		12	Df	+++
1052848-1	M		12	Df	+++
1049839-4	M		12	Df	+++
0896400-1	M	Tet.MoAβ to PrP.MoAβ/PS1 to PrP.HuAβ/PS1 (Df to Cr to Cr)	6	M	++
0896400-3	M		6	M	++
0896401-2	F		6	M	+
0896401-3	F		6	M	++
0904807-3	F		6	M	++
0970245_1	F	Tet.MoAβ to PrP.MoAβ/PS1 to APPsi (Df to Cr to Df)	11	Df	+++
0970245_2	F		11	Df	+++
0970245_3	F		11	Df	+++
0979027_1	M		11	Df	+++
0979027_2	M		11	Df	+++
0970245_1	F		11	Df	+++
0953016-1	F	Tet.MoAβ to PrP.MoAβ/PS1 to PrP.MoAβ/PS1 (Df to Cr to Cr)	12	Df	+++
0953016-2	F		12	Df	+++
0950580-1	F		12	Df	+++
0904803-3	M	PrP.HuAβ/PS1 to PrP.APPsi to PrP.HuAβ/PS1 (Cr to Df to Cr)	6	Cr	+
0904806-3	F		6	Cr	+
1015397-1	M		5	Cr	++
1015397-2	M		5	Cr	+
1015397-3	M		5	Cr	+
1015396-1	F		5	Cr	+
0914085_1	M	PrP.HuAβ/PS1 to PrP.APPsi to PrP.APPsi (Cr to Df to Df)	12	Df	+++
0914085_2	M		12	Df	+++
0914085_3	M		12	Df	+++
0914086_1	F		12	Df	+++
0914086_2	F		12	Df	++
0922186-2	M	PrP.MoAβ/PS1 to PrP.APPsi to PrP.HuAβ/PS1 (Cr to Df to Cr)	6	Cr	+
0922185-1	F		6	Cr	++
0904809-3	M		6	M	+
0904809-4	M		6	M	+
0904808-2	F		6	Cr	+
0904808-3	F		6	Cr	+
0901683_1	F	PrP.MoAβ/PS1 to PrP.APPsi to PrP.APPsi (Cr to Df to Df)	12	Df	+++
0901683_2	F		12	Df	+++
0957340_1	F		12	Df	+++

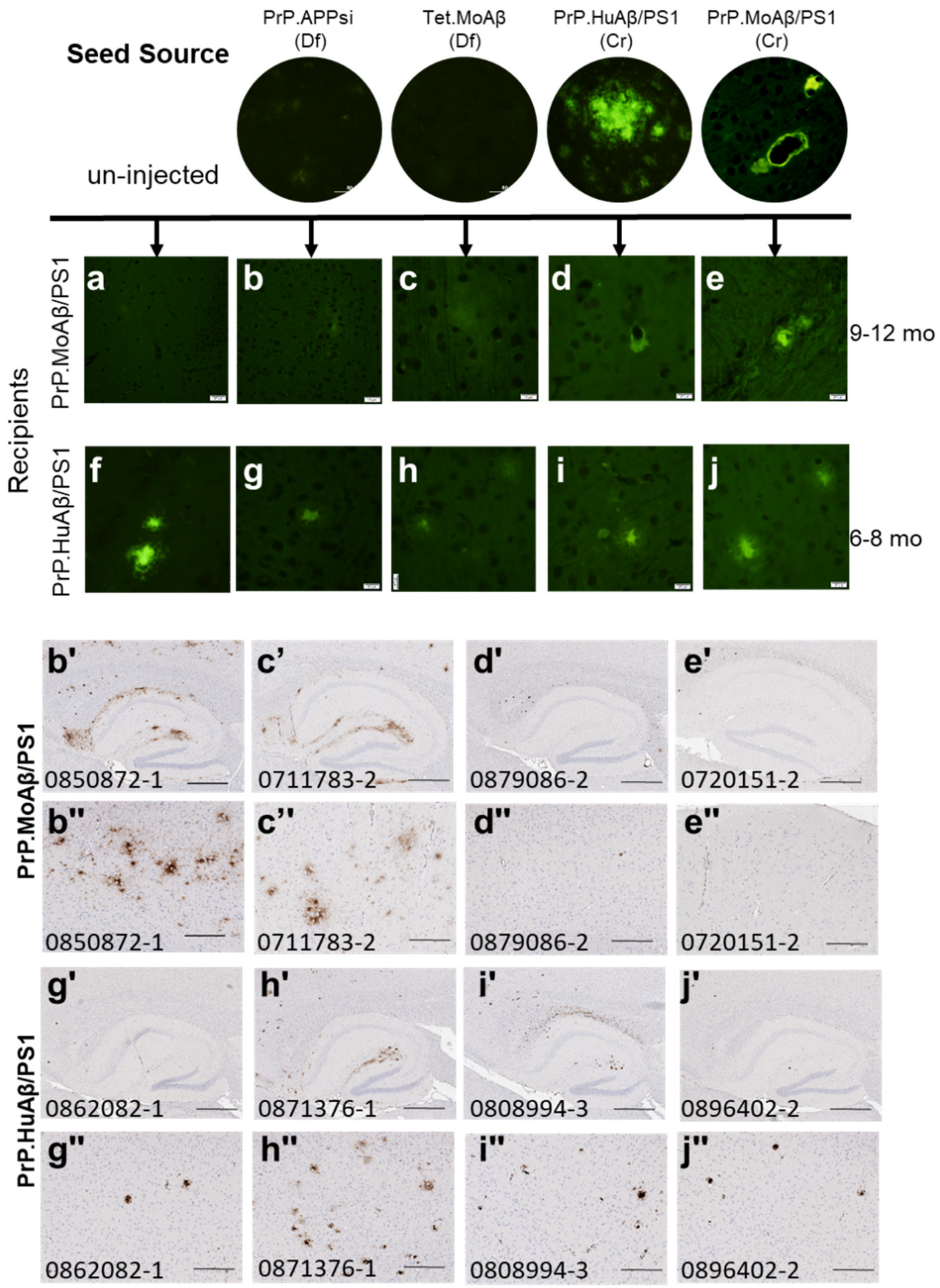

Figure S1. Representative images from tissue sections stained with Thio-S and Aβ antibodies for animals displayed in Figure 1. The layout of the figure is identical to that of Fig. 1 in the main text. The upper panels show high power images of Aβ deposit morphology from Thio-S silver staining in the brain of the seed source animal. The square images are from uninjected mice (a) and the brains of the recipients (b-j). All of these images are from cortex. b'-j'') Representative images or hippocampus and cortex of recipient mice. The labels and scale bars are the same as described in the legend of Figure 1 of the main text.

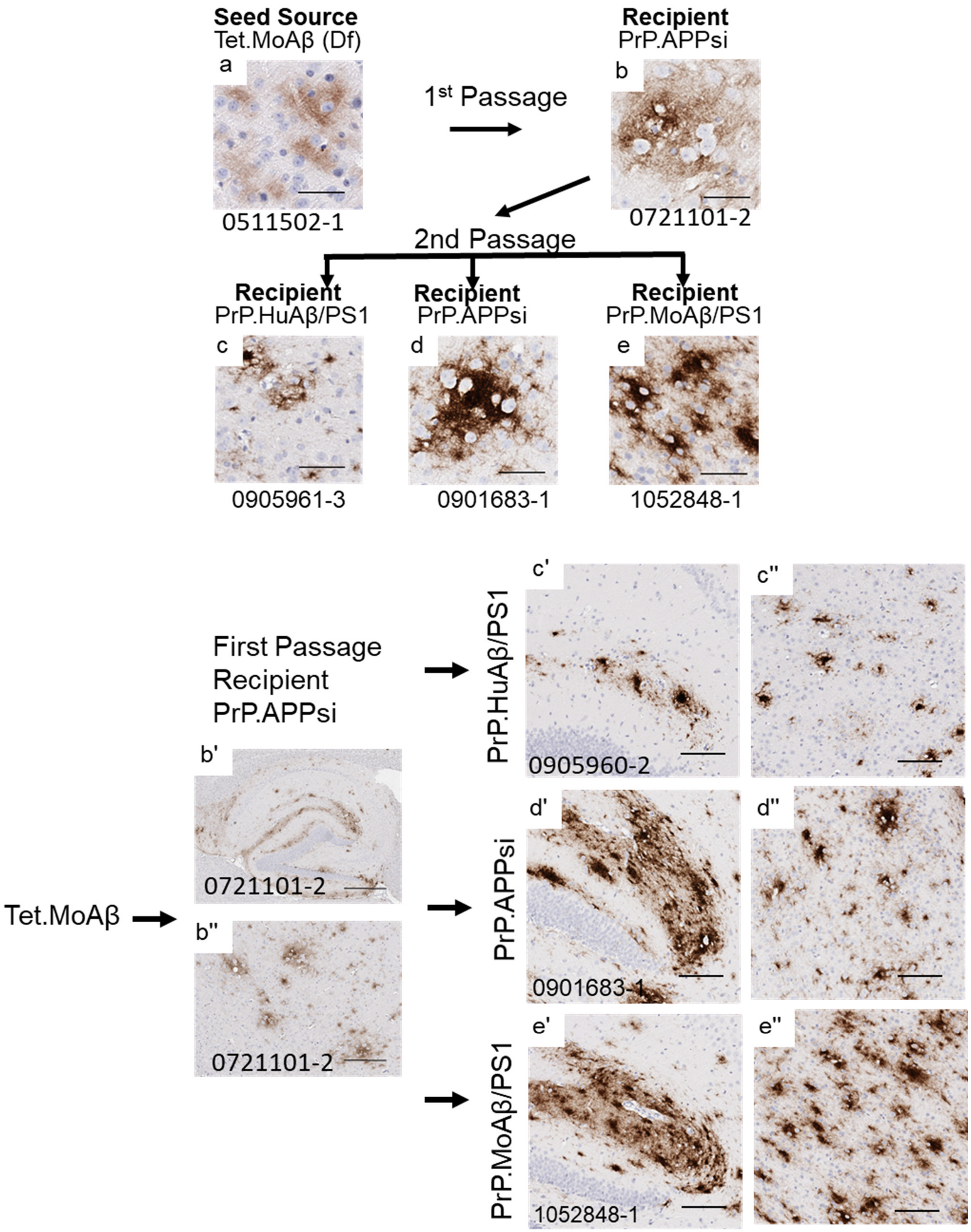

Figure S2. Representative images from tissue sections stained with Aβ antibodies for animals displayed in Figure 2. The layout of the figure, including labels and scale bars, is identical to the description of in the legend of Fig. 2 in the main text.

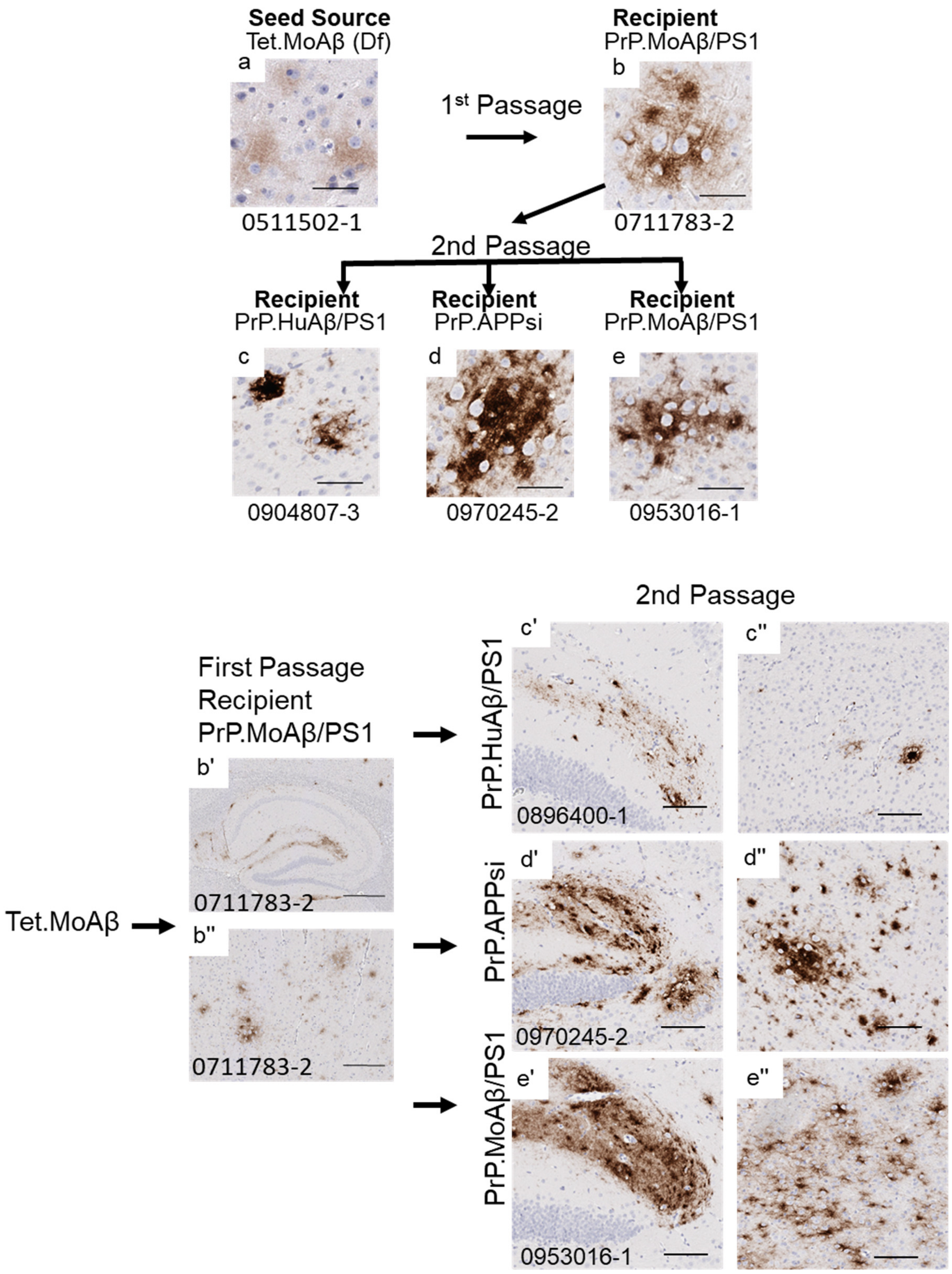

Figure S3. Representative images from tissue sections stained with Aβ antibodies for animals displayed in Figure 3. The layout of the figure, including labels and scale bars, is identical to the description of in the legend of Fig. 3 in the main text.

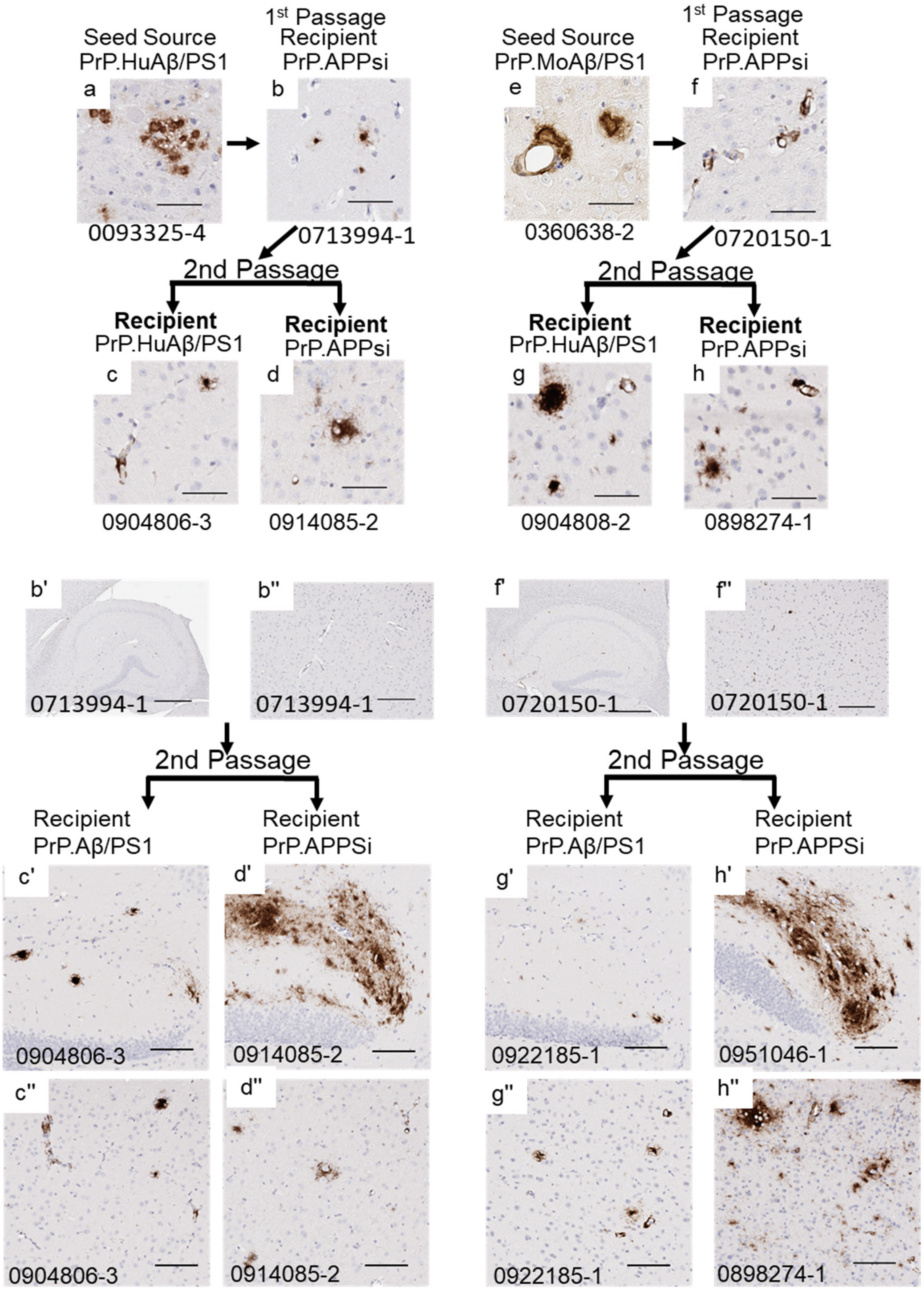

Figure S4. Representative images from tissue sections stained with Aβ antibodies for animals displayed in Figure 4. The layout of the figure, including labels and scale bars, is identical to the description of in the legend of Fig. 4 in the main text.
